# Associations of active commuting to school in childhood and physical activity in adulthood

**DOI:** 10.1038/s41598-023-33518-z

**Published:** 2023-05-11

**Authors:** Kaisa Kaseva, Irinja Lounassalo, Xiaolin Yang, Tuomas Kukko, Harto Hakonen, Janne Kulmala, Katja Pahkala, Suvi Rovio, Mirja Hirvensalo, Olli Raitakari, Tuija H. Tammelin, Kasper Salin

**Affiliations:** 1 Faculty of Sport and Health Sciences, University of Jyväskylä, Jyväskylä, Finland; 2 Faculty of Education Sciences, University of Helsinki, Helsinki, Finland; 3 School of Health and Social Studies, Jamk University of Applied Sciences, Jyväskylä, Finland; 4 Research Centre of Applied and Preventive Cardiovascular Medicine, University of Turku, Turku, Finland; 5 Centre for Population Health Research, University of Turku and Turku University Hospital, Turku, Finland; 6 Paavo Nurmi Centre & Unit for Health and Physical Activity, University of Turku, Turku, Finland; 7 Department of Clinical Physiology and Nuclear Medicine, Turku University Hospital, Turku, Finland

**Keywords:** Psychology, Health care, Risk factors

## Abstract

This study examined whether active commuting to school in childhood and adolescence predicted active commuting to work and overall physical activity (PA) in adulthood. Participants from the Young Finns Study (N = 2436) were aged 9–18 years in 1980 and followed up until 2018/2020. Their commuting modes to school were assessed with a self-reported questionnaire in 1980. Adulthood PA was assessed through self-reports regarding commuting modes to work (2001–2018), leisure-time physical activity (LTPA) (2001–2018), and objectively measured daily steps (2007–2018/2020). Associations between childhood commuting and adulthood PA were evaluated using regression analyses and multilevel models. Demographic, socioeconomic and environmental covariates were adjusted for in the analyses. Active commuting to school in childhood contributed favourably to LTPA in 2001 (B = .38, *p* < .001), in 2007 (B = .35, *p* < .001), and in 2018 (B = .28, *p* < .01). Active commuting in childhood was associated with higher number of daily aerobic steps (B = 299.00, *p* = .03) and daily aerobic steps during weekdays in 2011 (B = 312.15, *p* = .03). In 2018, active commuting associated favourably with daily aerobic steps (B = 370.42, *p* < .01), daily aerobic steps during weekdays (B = 347.65, *p* = .01), daily steps during weekends (B = 628.49, *p* = .02), and daily aerobic steps during weekends (B = 402.69, *p* = .03). Covariate adjustments attenuated the associations excluding the one between active commuting and LTPA in 2007 (B = .36, *p* = .01) and daily steps during weekends in 2018 (B = 782.25, *p* = .04). Active commuting to school in childhood might be one of the PA modes that contribute to PA in adulthood and is therefore encouraged to be promoted from an early age.

## Introduction

Non-communicable chronic diseases (NCD) are leading cause of global morbidity and mortality^1^. One of the most prominent risk factors for NCDs is physical inactivity^2,3^, referring to a failure to meet the recommended levels of physical activity (PA). At present, the positive effects of a physically active lifestyle are well-known, but more profound information on the facilitators of the development of such a lifestyle is still needed^1–4^. Although people’s level of interest in sports or leisure-time physical activities has been shown to vary, many might be motivated to integrate PA into their everyday routines^4^. Commuting by walking and cycling are some such activities^4^. Thus, it has been suggested that one potential way to prevent the development of NCDs with PA is investing in transport policies that enable physically active lifestyle choices, such as active commuting^1^.

Active commuting has been found to have numerous benefits on individual, social and environmental levels. Active commuting has been demonstrated to have a preventive effect on cardiovascular risk factors and outcomes, such as type 2 diabetes and myocardial infarction^5,6^. Furthermore, it is associated with a reduced risk of various cancers^6^. Active commuting has also been found to contribute favourably to physical fitness^7^ and body fat levels^8–10^. Research suggests that it is associated with increased levels of psychological wellbeing^11,12^, although the linkage has not been detected in all studies^13^. Furthermore, some evidence has indicated that commuting by walking or bicycling contributes to better work performance^14^ and that psychological and health benefits gained from commuting might mediate this association^15^. Regarding environmental benefits, walking, running, and bicycling reduce air pollution^16^, noise and risk exposures to traffic^17^.

In Finland, walking and cycling are common ways to commute to work and educational institutions^18,19^, and over 80% of 10- to 16-year-old students walk or cycle to school^19^. However, active commuting habits tend to decline along with age, with the decline often starting between ages 12 and 15^20^. The age-related decline in active commuting has been shown to be a global phenomenon^19,21,22^. Additionally, the number of passive commuters (public transport or car) is nearly 70% in Finland^23^. In order to promote lifelong physical activity^24^, understanding the contributors that affect the commitment to different types of physical activities over time is important.

It has been shown that health behaviours, including different types of physical activities, begin to modify at an early age^24,25^. Despite the recognized decline in active commuting (e.g.^18^), previous tracking studies on PA have also shown that PA habits are relatively stable during all phases of life, especially in men^24^. The correlations have been shown to be lowest during transitional periods^24^. Additionally, it has been discussed that inactive behaviours accumulate over the transition from adolescence to young adulthood^26^. Some rare studies have demonstrated that active commuting in childhood might not predict total weekly PA in later life^27^.

Child-friendly communities and the overall atmosphere of social environments have also been shown to be associated with the likelihood of active commuting^28^. Seasonal variation has also influenced PA, with individuals being less active during wintertime^26^. Specifically, seasonal variation has been shown to affect girls’ active commuting and children living in rural areas more^29^. There might also exist socioeconomic inequalities in the ways of commuting^30^. It has been previously speculated that transport cultures and infrastructures may play a role in the development of commuting habits, e.g. children who are driven even short distances might not appreciate active commuting as an adult^14,28^. Furthermore, transitional periods in life can affect the interest to engage in physical activities^24,31^, but also offer novel opportunities to integrate PA into daily life (e.g. walking and cycling to schools, universities and workplaces)^32^.

To promote active commuting, it is necessary to understand whether and how active commuting in childhood is related to commuting as well as to other physical activities in adult age. Some past evidence on active commuting’s health benefits derives only from cross-sectional studies^13^. There also exists a need for studies assessing the change and stability of PA over time and information on to what extent engaging in specific types of sports tracks into young adulthood^33^. Furthermore, it is essential to pay attention to demographic, socioeconomic and environmental correlates that potentially affect the linkages between physical activities in childhood and adulthood^34^. Overall, more careful evaluation regarding the roles of different PA types, including active commuting, is needed in understanding the development and maintenance of a physically active lifestyle^34^.

The study participants were from the ongoing, community-based, observational Cardiovascular Risk in Young Finns Study. The present study aims to (1) examine whether active commuting in childhood is associated with active commuting in adulthood, (2) assess whether active commuting in childhood is associated with adulthood PA, including self-reported leisure time physical activity (LTPA) and objectively assessed steps, and (3) whether the potential associations are robust when adjusting for a set of demographic, socioeconomic and environmental covariates.

## Methods

### Study design and population

The study participants were from the ongoing community-based Cardiovascular Risk in Young Finns Study, which consists of six cohorts born in 1962, 1965, 1968, 1971, 1974 and 1977^35^. The original sample consisted of 3596 (1764 males and 1832 females) children and adolescents (83.20% of those invited). To attain a representative sample, Finland was divided into five areas based on the locations of universities with medical schools (Helsinki, Kuopio, Oulu, Tampere, and Turku), and the subjects were randomly selected based on their social security numbers from nearby urban and rural areas. Informed consent was obtained from participants or legal guardian. The study was approved by the Ethics Committee of the Hospital District of Southwest Finland. It was conducted in accordance with the Declaration of Helsinki (revised in 1983), and it complied with American Psychological Association’s ethical guidelines.

After the baseline assessments, the sample has been followed in 1983, 1986, 1989, 1992, 2001, 2007, 2011 and 2018–2020. In the current study, participants’ commuting mode to school was assessed in 1980 at participants’ age of 9, 12, 15 and 18. Data were restricted to children and adolescents at these ages, because they were participating in compulsory education in Finland and were thus commuting to school. Children who were 3 or 6-year-olds at the baseline were not included in the present study, and only those 18-year-olds who were still participating in schoolwork (e.g., vocational or high school) during 1980 responded to our question regarding their commuting to school. Adulthood physical activity was assessed in 2001 (participants aged 30–39), in 2007 (participants aged 36–45), in 2011 (participants aged 40–49), and 2018 (participants aged 47–56). Based on the previous studies on sample attrition, no systematic selection bias has been reported regarding self-reported or device-measured PA^36–38^.

### Commuting in childhood

Participants self-reported their mode of travelling to school (assessed in 1980). The original variable reflecting commuting was coded as 1 = own car or carpool, 2 = walking, 3 = cycling, 4 = other mode (e.g. using public transport). The participants travelling to school by walking or cycling were categorised as active commuters. In contrast, those who were driven to school by car or public transportation were coded as inactive commuters. Within the baseline (1980), the potential seasonal variation (e.g. whether participants’ commuting modes differed during summer and winter seasons) was not addressed.

### Self-reported PA in adulthood

Participants’ commuting mode to workplace was examined in adulthood (2001, 2007, 2011 and 2018) using the following response options: 1 = own car or carpool, 2 = public transportation, 3 = walking, 4 = cycling. The participants commuting to work by walking or cycling were categorised as active commuters, whereas those using cars or public transportation were categorised as passive commuters. All follow-ups included a separate question assessing commuting in summer and wintertime (2001–2018).

Participants’ leisure-time PA (LTPA) was addressed through five questions as follows: (1) How much breathlessness and sweating do you experience when you engage in PA and sport? (2) How often do you engage in rigorous PA? (3) How many hours per week do you engage in rigorous PA? (4) How much time do you usually spend in a PA session? (5) Do you participate in organized PA? The responses were rated using a scale ranging from 1 to 3. A sum score (PA index) of the responses was created for each participant in each study year (2001, 2007, 2011 and 2018) (Supplementary Table 1) to reflect the participants’ LTPA. The PA index values ranged between 5 and 15 with higher scores indicating higher levels of PA (Supplementary Table 1). PA indices were used as reflections of participants’ LTPA.

### Pedometer and accelerometer-measured PA

Number of steps has proven to be a feasible measure for monitoring PA^39^. Within the present study, participants’ steps per day were measured by motion sensors using the Omron Walking Style One pedometer (HJ-152R-E; Omron, Kyoto, Japan) in 2007–2008 and 2011–2012 and the triaxial hip-worn ActiGraph accelerometer (GT3X+ and wGT3X+; ActiGraph Pensacola, FL) in 2018–2020. The detailed descriptions regarding the application of pedometers and accelerometers are reported elsewhere^38^. Previous studies have shown that at least 360 min should have been measured through a device for a valid day^24^. Furthermore, a four-day measurement has proven to be a reliable method when investigating PA^40^. Thus, the pedometer data were considered valid if the participant reported wearing the device for all waking hours per day on at least four days of seven consecutive days. Regarding accelerometers, a valid day was defined as recording at least 600 min of measured wear time between 07:00 and 23:00. Analyses of PA were restricted to participants who provided at least four days (three weekdays + one weekend day) of valid accelerometer data. In general, the instruments and protocol for data collection have been shown to provide valid and reliable step-counting information, and the step counts from both devices are highly correlated (r ≥ 0.90)^41^.

In the present study, the daily number of steps and aerobic steps on average, as well as daily steps and daily aerobic steps separately during weekdays and weekend days in the years 2007–2018/2020 were examined as outcome measures. Aerobic steps were accumulated in bouts of at least 10 min when at least 60 steps per minute were counted.

### Covariates

Participants’ age and sex (1980) were controlled for in the analyses. Participants’ own and their parents’ education (1983) and income levels (1980) were used as indicators of socioeconomic status in participants’ childhood and adulthood. Information on parents’ educational background was collected from both mothers and fathers [(1 = primary school or below, 2 = secondary education (high school, secondary education institution), 3 = tertiary education (university level)]. Family's income level was studied using an 8-point scale [1 =  < 15,000 marks (€2523), 8 =  > 100,000 marks (€16,819)]. Furthermore, participants’ childhood living area (1 = city centre, 2 = suburb, 3 = rural community, 4 = dispersed settlement area) was controlled for in the analyses.

Participants’ latest educational level was also adjusted for in the examinations. In 2001–2011, the educational level was coded as 1 = secondary educational institution/ level, 2 = lower academic studies/ degree (some academic studies or bachelor’s degree), 3 = upper academic studies/ degree (master’s degree or higher). In 2018, the coding regarding participants’ education was as follows 1 = secondary educational level, 2 = some academic studies, 3 = academic degree (bachelor’s degree, master’s degree or higher). In 2001, the information on participants’ income level was not available. In 2007, participants’ income level was studied using an 8-point scale [(1 =  < €10,000, 8 =  > €70,000]. In 2011, participants’ income was assessed through a 13-point scale (1 =  < €5000, 13 =  > €60,000). In 2018, participants’ income level was assessed through a 21-point scale (1 =  < €5000, 21 =  > €100,000). Furthermore, the distance (km) from participants’ home to their workplace was measured in the year 2018.

In 2001–2011, the living area was coded through a 4-point scale (1 = city centre, 2 = suburb, 3 = rural community, 4 = dispersed settlement area). In 2018 the inquiry about living area was lacking, and it was replaced with the information on participants’ living area in 2011 in the adjusted analyses.

### Statistical analyses

The associations between childhood (1980) and adulthood commuting (2001–2018) were studied using logistic regression analyses, and the associations between childhood commuting and other indicators of PA through linear regression models. The analyses were adjusted for participant’s age, sex, mother’s and father’s education levels (1983), family’s income (1980), childhood living area (1980), participants’ own educational status (2001–2018), living area (2001–2011), income level (2007–2018), and distance (km) from the participant’s home to workplace (2018). Regarding participants’ educational status, income levels and living area, the newest information available was controlled for in the regression models. In case the unadjusted models’ significant results changed when all the covariates were adjusted for in these models, we controlled for each covariate separately in the models’ first step, followed by the main predictor, to evaluate which specific covariate affected the associations. As supplementary analyses, we used multilevel modelling to evaluate the associations longitudinally, as well as to perform attrition analyses. Detailed information on the variables and codings are presented in Supplementary Table 4, and in the results section.

The longitudinal logistic and linear regression analyses were performed using SPSS statistical software (version 28). The software R (version 4.2.1) and lmerTest package were used in the multilevel modelling^42^.

### Ethics approval

The study was approved by the Ethics Committee of the Hospital District of Southwest Finland. Informed consent was obtained from participants or legal guardian.

## Results

**Figure 1:**
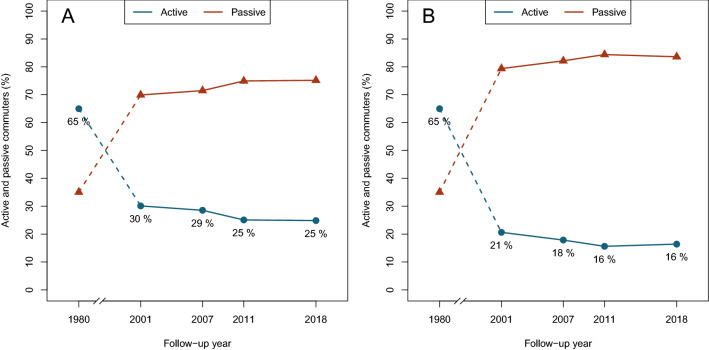
Proportions of active and passive commuters from 1980 to 2018. The graphs represent participants' commuting to school (**A** & **B**), commuting to work in the summertime (**A**), and commuting to work in the wintertime (**B**).

The descriptive statistics of the sample are presented in Supplementary Tables 1, 2, 3 and Fig. 1. Childhood commuting modes did not predict participants’ adulthood commuting (all *p* values > 0.05) (see Tables 1 and 2). Active commuting in childhood in 1980 contributed favourably to LTPA in adulthood in 2001 (B = 0.38, *p* < 0.001, 95% CI: 0.18 to 0.58), in 2007 (B = 0.35, *p* < 0.001, 95% CI: 0.15 to 54), and in 2018 (B = 0.28, *p* = 0.01, 95% CI: 0.06 to 0.50). Active commuting in childhood was associated with a higher number of daily aerobic steps (B = 299.00, *p* = 0.03, 95% CI: 28.73 to 568.80) and daily aerobic steps during weekdays in 2011 (B = 312.15, *p* = 0.03, 95% CI: 37.78 to 586.53). Furthermore, active commuting was associated with daily aerobic steps (B = 370.42, *p* < 0.01, 95% CI: 117.15 to 623.69), daily aerobic steps during weekdays (B = 347.65, *p* = 0.01, 95% CI: 96.53 to 598.76), daily steps during weekends (B = 628.49, *p* = 0.02, 95% CI: 120.93 to 1136.04), and daily aerobic steps during weekends (B = 402.69, *p* = 0.03, 95% CI: 32.96 to 772.42) in 2018/2020 (Table 3).

**Table 1:** Childhood commuting to school in 1980 predicting adulthood commuting to work in 2001–2018 (N = 904–1263).

Variables	B	SE	p	OR [Exp.(B)]	95% CI (lower)	95% CI (upper)	Nagelkerke R^2^
Commuting during summer, 2001	0.19	0.12	0.11	1.21	0.96	1.52	0.00
Commuting during winter, 2001	−0.00	0.13	0.97	1.00	0.77	1.29	0.00
Commuting during summer, 2007	0.10	0.13	0.45	1.10	0.86	1.41	0.00
Commuting during winter, 2007	0.10	0.15	0.52	1.10	0.83	1.47	0.00
Commuting during summer, 2011	0.12	0.14	0.38	1.13	0.86	1.47	0.00
Commuting during winter, 2011	−0.06	0.16	0.73	0.95	0.69	1.30	0.00
Commuting during summer, 2018	−0.02	0.14	0.89	0.98	0.74	1.30	0.00
Commuting during winter, 2018	0.02	0.17	0.93	1.02	0.73	1.42	0.00

**Table 2:** Childhood commuting to school in 1980 predicting adulthood commuting to work in 2001–2018 adjusting for covariates (N = 647–924)^1,2^.

Variables	B	SE	p	OR [Exp. (B)]	95% CI (lower)	95% CI (upper)	Nagelkerke R^2^
Commuting to workplace during summer, 2001	0.11	0.18	0.54	1.12	0.78	1.60	0.09
Commuting to workplace during winter, 2001	−0.05	0.20	0.80	0.95	0.64	1.41	0.06
Commuting to workplace during summer, 2007	−0.10	0.20	0.61	0.91	0.62	1.33	0.17
Commuting to workplace during winter, 2007	0.04	0.22	0.85	1.04	0.68	1.60	0.15
Commuting to workplace during summer, 2011	−0.01	0.21	0.98	1.00	0.66	1.50	0.12
Commuting to workplace during winter, 2011	−0.03	0.26	0.92	0.98	0.59	1.63	0.14
Commuting to workplace during summer, 2018	−0.58	0.31	0.06	0.56	0.31	1.03	0.60
Commuting to workplace during winter, 2018	−0.35	0.38	0.35	0.70	0.34	1.47	0.64

^1^The analyses were adjusted for participants’ age, sex, parents’ educational status (in childhood), family’s income, childhood living area, participants’ own educational status, income, and adulthood living area. The newest information regarding participants’ educational status, income, and adulthood living area were adjusted for in the models. The associations between childhood and adulthood commuting in 2018 were also adjusted for the variable reflecting the distance (km) from home to workplace.

^2^Regarding the categorical covariates, first category was used as a reference value. The categories are presented in the Supplementary Table 3.

**Table 3:** Childhood commuting to school in 1980 predicting overall physical activity in 2001–2018/2020 (N = 935–1616).

Variables	B	SE	β	*p*	95% CI (lower)	95% CI (upper)	Adjusted R^2^
LTPA, 2001	0.38	0.10	0.09	< 0.001	0.18	0.58	0.01
LTPA, 2007	0.35	0.10	0.09	< 0.001	0.15	0.54	0.01
LTPA, 2011	0.09	0.11	0.02	0.43	−0.13	0.30	0.00
LTPA, 2018	0.28	0.11	0.07	0.01	0.06	0.50	0.00
Pedometer measurements							
Year 2007							
Daily steps (week)	18.32	194.94	0.00	0.93	−364.21	400.85	−0.00
Daily aerobic steps (week)	123.20	136.08	0.03	0.37	−143.84	390.23	0.00
Daily steps (weekdays)	65.51	211.38	0.01	0.76	−349.30	480.31	−0.00
Daily aerobic steps (weekdays)	123.21	138.61	0.03	0.37	−148.80	395.21	0.00
Daily steps (weekends)	223.00	238.56	0.03	0.35	−245.16	691.14	0.00
Daily aerobic steps (weekends)	205.88	195.11	0.03	0.29	−177.00	588.76	0.00
Year 2011							
Daily steps (week)	206.41	203.81	0.03	0.31	−193.51	606.32	0.00
Daily aerobic steps (week)	299.00	137.62	0.07	0.03	28.73	568.80	0.00
Daily steps (weekdays)	234.46	219.26	0.03	0.29	−195.79	664.71	0.00
Daily aerobic steps (weekdays)	312.15	139.83	0.07	0.03	37.78	586.53	0.00
Daily steps (weekends)	182.30	252.71	0.02	0.47	−313.61	678.21	0.00
Daily aerobic steps (weekends)	376.13	199.77	0.06	0.06	−15.88	768.13	0.00
Accelerometer measurements Year 2018–2020							
Daily steps (week)	241.00	212.11	0.04	0.26	−175.28	657.27	0.00
Daily aerobic steps (week)	370.42	129.06	0.09	0.00	117.15	623.69	0.01
Daily steps (weekdays)	68.10	229.07	0.01	0.77	−381.45	517.66	−0.00
Daily aerobic steps (weekdays)	347.65	127.96	0.09	0.01	96.53	598.76	0.01
Daily steps (weekends)	628.49	258.62	0.08	0.02	120.93	1136.04	0.01
Daily aerobic steps (weekends)	402.69	188.39	0.07	0.03	32.96	772.42	0.00

Regarding the self-reported adulthood physical activity, adjusting for the covariates attenuated the associations, excluding the one with active commuting in childhood and LTPA in 2007 (B = 0.36, *p* = 0.01, 95% CI: 0.09 to 0.63) (Table 4). With respect to childhood commuting and LTPA in 2001, none of the covariates that were adjusted separately for in the model attenuated the association. Thus, we concluded that controlling for the combination of all covariates (participants’ age, sex, parents’ educational statuses, childhood living area, participants’ own educational status, and adulthood living area) in the same model attenuated the association (*p* > 0.05). The association between childhood commuting and LTPA in 2018 was attenuated after adjusting for parents’ educational statuses, as well as adulthood income (*p* > 0.05) (Table 4).

**Table 4:** Childhood commuting to school in 1980 predicting overall physical activity in 2001–2018/2020 adjusting for covariates (N = 497–948)^1^.

Variables	B	SE	β	*p*	95% CI (lower)	95% CI (upper)	Adjusted R^2^
Self-reported LTPA							
LTPA, 2001^2^	0.15	0.14	0.04	0.29	−0.13	0.43	0.03
LTPA, 2007	0.36	0.14	0.09	0.01	0.09	0.63	0.04
LTPA, 2011	−0.14	0.15	−0.04	0.36	−0.44	0.16	0.04
LTPA, 2018^3^	0.14	0.17	0.03	0.43	−0.20	0.48	0.06
Pedometer measurements							
Year 2007							
Daily steps (week)	148.71	279.09	0.02	0.59	−399.34	696.75	0.00
Daily aerobic steps (week)	14.16	186.59	0.00	0.94	−352.25	380.57	0.07
Daily steps (weekdays)	353.80	300.54	0.05	0.24	−236.42	944.01	0.01
Daily aerobic steps (weekdays)	72.10	188.74	0.02	0.70	−298.54	442.74	0.07
Daily steps (weekends)	−49.21	334.67	−0.01	0.88	−706.44	608.03	0.01
Daily aerobic steps (weekends)	−8.88	270.13	−0.00	0.97	−539.39	521.61	0.03
Year 2011							
Daily steps (week)	83.65	279.09	0.01	0.76	−464.42	631.72	0.00
Daily aerobic steps (week)^4^	108.76	187.21	0.02	0.56	−258.88	476.40	0.05
Daily steps (weekdays)	184.49	308.72	0.03	0.55	−421.79	790.77	0.00
Daily aerobic steps (weekdays)^5^	174.91	198.07	0.04	0.38	−214.07	563.87	0.04
Daily steps (weekends)	−89.44	327.80	−0.01	0.79	−733.22	554.34	0.01
Daily aerobic steps (weekends)	21.59	254.46	0.00	0.93	−478.15	521.33	0.03
Accelerometer measurements							
Year 2018–2020							
Daily steps (week)	396.21	320.55	0.06	0.22	−233.61	1026.04	0.02
Daily aerobic steps (week)^6^	216.11	190.19	0.05	0.26	−157.59	589.80	0.04
Daily steps (weekdays)	220.87	349.20	0.03	0.53	−465.25	906.99	0.02
Daily aerobic steps (weekdays)^7^	118.93	186.65	0.03	0.52	−247.81	485.68	0.05
Daily steps (weekends)	782.25	376.25	0.10	0.04	42.96	1521.54	0.02
Daily aerobic steps (weekends)^8^	425.52	282.35	0.07	0.13	−129.26	980.30	0.02

^1^The analyses were adjusted for participant’s age, sex, parents’ educational status, family’s income, childhood living area, participants’ own educational status, income and adulthood living area. In the analyses within which the outcome was assessed in 2018, the distance from participant’s home to workplace (km) was also adjusted for. The newest information available regarding participants’ educational status, income, and adulthood living area were adjusted for in the models.

^2^The association between childhood commuting and LTPA 2001 was attenuated after adjusting for all the covariates in the same model (*p* > 0.05).

^3^The association between childhood commuting and LTPA in 2018 attenuated after adjusting for mother’s and father’s educational status, as well as adulthood income (*p* > 0.05).

^4^The association between childhood commuting and daily aerobic steps attenuated after adjusting for mother’s and father’s educational status, childhood living area, participant’s educational status, and adulthood living area (*p* > 0.05).

^5^The association between childhood commuting and daily aerobic steps during weekdays attenuated after adjusting for mother’s and father’s educational status, childhood living area, and adulthood living area (*p* > 0.05).

^6^The association between childhood commuting and daily aerobic steps attenuated after adjusting for all the covariates at the same model (*p* > 0.05).

^7^The association between childhood commuting and daily steps during weekdays in 2018/2020 attenuated after adjusting for father’s educational status (*p* > 0.05).

^8^The association between childhood commuting and daily aerobic steps during weekends attenuated after adjusting for participants’ age, father’s educational status, and participant’s educational status (*p* > 0.05).

All the associations between childhood commuting and objectively assessed physical activity were attenuated after covariate adjustments, excluding the one with childhood commuting and daily steps during weekends 2018/2020 (B = 782.25, *p* = 0.04, 95% CI: 42.96 to 21.54). The association between commuting and daily aerobic steps in 2011 attenuated after adjusting for the educational statuses of the parents, childhood living area, participant’s educational status, and adulthood living area (*p* > 0.05). The linkage between commuting and daily aerobic steps during week days in 2011 was attenuated after controlling for parents’ educational statuses, childhood living area, and adulthood living area (*p* > 0.05) (Table 4). Regarding the years 2018/2020, none of the covariates that were separately controlled for in the analyses attenuated the association between childhood commuting and daily aerobic steps. Thus, we concluded that controlling for the combination of all the covariates attenuated the association (*p* > 0.05) (Table 4). The linkage between childhood commuting and daily steps during weekdays disappeared after adjusting for father’s educational status (*p* > 0.05). The association between the childhood commuting and daily aerobic steps during weekends was attenuated after adjusting for participants’ age, father’s educational status, and participant’s educational status (*p* > 0.05).

We also re-tested the significant associations between childhood commuting and adulthood physical activity within datasets that were restricted to those participants who had delivered information on all the covariates within each study year (N = 497–948). Within these analyses, childhood commuting predicted LTPA in 2001 (B = 0.32, *p* = 0.02, 95% CI: 0.06 to 0.58), and in 2007 (B = 0.39, *p* < 0.01, 95% CI: 0.13 to 0.64). We also found marginally significant associations between childhood commuting and daily aerobic steps in 2018/2020 (B = 321.86, *p* = 0.08, 95% CI: −35.54 to 679.25), and daily aerobic steps during weekends in 2018/2020 (B = 464.69, *p* = 0.08, 95% CI: −61.84 to 991.07).

In the framework of multilevel modelling, it is essential to code the variables in a coherent manner across the study years. Within the present study, the availability and consistency of the covariates and outcome variables are presented in the Supplementary Table 4. Most of the variables were measured concurrently across the study years, although some inconsistencies were present. Due to these issues, participants’ income level in 2001 was supplemented by the data of next the follow-up year, 2007. Data on income was re-classified to obtain an identical covariate across the study years. Furthermore, adulthood living area in 2018 was supplemented with the information from the previous follow-up year, 2011. Commuting distance from home to work was available only for the latest follow-up, 2018, and it was therefore omitted from the modelling. Device-based measurements were not available in 2001. Therefore, outcome variables of steps and aerobic steps were combined only for the three last follow-ups. Also, the follow-up year was added to the models as an additional covariate. This addition not only confronts properly the longitudinal structure of new set, but also accounts for the changes in measurement devices during the follow-ups. Participants’ ID was used as a random intercept and the fixed effect part was modelled as in the separate analysis of four (three, for device-based outcomes) follow-ups. The covariate adjustments were performed similarly as in each logistic and linear regression analysis, with the exceptions listed in the Supplementary Table 4 and the sentences above.

The results from the unadjusted mixed models demonstrated that active commuting in childhood was not associated with adulthood commuting or daily steps (all *p*’s > 0.05). Childhood commuting was associated with LTPA in adulthood (B = 0.28, *p* < 0.001, 95% CI: 0.14 to 0.43) and daily aerobic steps (weekly average B = 225.9, *p* = 0.02, 95% CI: 38.2 to 413.5; weekends B = 317.0, *p* = 0.02, 95% CI: 41.0 to 559.8; weekdays B = 216.5, *p* = 0.03, 95% CI: 31.5 to 401.4) (Supplementary Table 5). All the associations attenuated after adjusting for the covariate pattern (all *p* values > 0.05) (Supplementary Table 6).

Within the attrition analyses, we studied how the baseline measurements (1980, 1983) were associated with continuing in the study through the follow-up years. Regarding the questionnaire data, older persons were more likely to continue in the study in the follow-ups (OR = 1.19,* p* < 0.001). Men were more likely to drop out from the study than women (OR = 0.48, *p* < 0.001). Active commuters in the childhood were more likely to stay within the follow-ups than the passive ones (OR = 2.05, *p* < 0.001). Highly educated mothers were more likely to drop out than mothers with lower education levels (OR = 0.36, *p* = 0.02). Fathers with secondary education were more likely to drop out than fathers with lower education level (OR = 0.38, *p* < 0.001). Families with higher income levels were more likely to stay within the study than the families with lower income levels (ORs 2.76–6.15 for income levels 4–8, all *p* values < 0.05). During the years 2007–2018, more participants discontinued comparing to follow-up in 2001 (ORs 0.23–0.39, all *p* values < 0.001). For further details, see Supplementary Tables 7–8.

## Discussion

This study examined whether active commuting to school in childhood was associated with active commuting to work in adulthood within a population-based, cohort study conducted among Finns. The study also assessed whether active commuting in childhood was associated with self-reported commuting to work and LTPA in adulthood, as well as with pedometer- and accelerometer-measured daily steps. Finally, the study tested whether the relationships were robust to covariate adjustments, as well as which specific covariates attenuated the detected associations.

Our main findings, which we based on the longitudinal logistic and linear regression analyses, indicated that commuting to school in childhood was not associated with the modes of adulthood commuting. We found, however, associations between childhood commuting and LTPA in participants’ early and middle adulthood (years 2001, 2007, and 2018), as well as linkages between childhood active commuting to school and steps in adulthood (years 2011 and 2018). Although these results were in the same direction with the ones in unadjusted multilevel models, no longitudinal linkages remained significant after covariate adjustments in these models. The results provide partial support for previous studies demonstrating the linkages between PA over time^24,43^, suggesting that the potential tracking of PA over different developmental stages from childhood to adult age might be related to the type of PA.

After adjusting for the analyses for participants’ age, sex, parents’ educational levels in participant’s childhood, family’s income, childhood living area, participants’ own educational status, income, and adulthood living area, and distance (km) from home to work (examined in the regression analyses in 2018) the significant associations were diluted, excluding the one with childhood commuting and adulthood LTPA assessed in 2007 and the one with childhood commuting and daily steps at weekends 2018/2020. In principal, the socioeconomic factors such as education, as well as environmental aspects such as living area seemed to be the most common factors that attenuated the associations. Regarding the longitudinal models, adjusting for all covariates diluted the associations. Nevertheless, as we were not able to apply all the covariates in their most accurate form in these supplementary analyses, we have carefully based our main conclusions on the linkages between childhood commuting and various PA types in the logistic and linear regression analyses.

The causes for associations between childhood commuting and adulthood LTPA can be individual as well as social and environmental. Regarding the linkage between childhood commuting and adulthood LTPA in 2007 and steps in 2018/2020, supportive social environments can facilitate the commitment to various physical activities over the life course^31^. The linkage between childhood commuting and adulthood LTPA can also be related to changes in living conditions’ during the life course^31^. Therefore, the tendencies to move (e.g. using a bicycle) may have been modified according to what types of opportunities for PA people perceive as available in their living areas. This highlights the need to keep on investing in such transport policies that enable attractive physically active lifestyle choices for all age groups^1^. One’s genetic background may be associated with a person’s interest and maintenance of physical activities^44^. Genetic background also forms a basis for one’s temperamental and personality characteristics, which can determine, to a certain degree, to what extent a person gets interested in various physical activities during the life course^45,46^. Some evidence has also indicated that commuting by walking or bicycling in adolescence contributes to psychological wellbeing^12^. Due to memory traces relating to favourable psychological states, people might search for similarly psychologically fulfilling experiences from different physical activities also in adulthood.

The socioeconomic conditions in participants’ childhood and adulthood had one of the strongest confounding effects on the detected associations. The attenuation of many of the associations due to parents’ educational levels, for instance, aligns with previous work stating that socioeconomic factors contribute to physical activities^30,47^. As it has been previously shown that low socioeconomic status contributes to unhealthy lifestyle choices^47^, it is possible that physical activities have not been equally valued among this study’s families. On the other hand, it is also worth considering that if the participants’ parents have had the financial opportunity and will to drive their children even short distances, the offspring may not appreciate active commuting as an adult^28^. Overall, there is a need to study more profoundly the role of socioeconomic factors in relation to the development of physically active lifestyles^48^.

The present study indicated that childhood commuting to school contributed to self-reported LTPA and objectively assessed steps in adulthood. However, the associations were not consistent up to middle age. The findings suggest that active commuting to school in childhood might be one of the PA modes that contribute to physical activity in adulthood. These findings are in the same direction with previous evidence from the same dataset highlighting the importance of ambulatory activity in the maintenance of an active lifestyle^38^.

### Limitations

The adjusted covariates were not identical across the years. All the covariates that reflected participants’ living conditions were not assessed in 1980 which may have contributed to the sample size’s reduction. We were not able to control for the distance (km) from participants’ childhood home to their school. We had information on the distance (km) from participant’s home to workplace only from the year 2018. Regarding future studies on childhood commuting, these potential confounding factors should be controlled for more comprehensively^49^. Our study is also observational, meaning that the interpretations of the results should be performed with caution. In order to gain supportive evidence regarding the causality between the examined phenomena, experimental research would be needed. Although the previous studies on sample attrition have given supportive evidence that no systematic selection bias exists regarding self-reported or device-measured PA^36–38^, we cannot exclude the possibility of selection bias within the present study especially regarding the demographic and socioeconomic factors. Participants who have travelled to school or work by train, tram or bus may have cycled or walked to get the public transport, but the amount of PA undertaken during these moments remains unknown. Travel modes are also changing. One or two more response options could have been included in the latest data collection, such as skateboarding and using a scooter. Assessing the participants’ potential hours per week of remote working days would also be beneficial.

### Strengths

The strength of the study is the population-based, large dataset with multiple cohorts. Furthermore, we were able to estimate the outcome variables using both self-reports and device-based measurements of PA. An extensive set of potential confounders was controlled for. We applied logistic and linear regression analyses, as well as longitudinal data modelling techniques which have also been utilized in the previous examinations^50^. To our knowledge, the present study is one of the first to have assessed the contribution of childhood commuting activity to school to a variety of indicators of adulthood PA over 40 years.

## Conclusions

Active commuting to school in childhood might be one of the PA modes that contribute to PA in adulthood and is therefore encouraged to be promoted from an early age.

## Supplementary Information

Supplementary Information.

## Data Availability

Restrictions apply to the availability of the data. The data presented in this study may be assessed by request from Prof. Olli Raitakari.
