# Supplementary material for: Associations of active commuting to school in childhood and physical activity in adulthood
Source: Sci Rep. 2023 May 11;13:7642. doi: 10.1038/s41598-023-33518-z (PMC10175542; doi:10.1038/s41598-023-33518-z)
Supplement: Supplementary file 1 — Supplementary Information. [file 41598_2023_33518_MOESM1_ESM.docx]

**Supplementary Table 1**. Descriptive statistics of the variables reflecting commuting to school in childhood (1980) and to work in adulthood (2001-2018) (N = 1232-2352)

| Variable | N (%) |
| --- | --- |
| Childhood commuting 1980  Passive commuters  Active commuters | 2352 (100.00)  824 (35.00)  1528 (65.00) |
| Commuting summer 2001  Passive commuters  Active commuters | 1618 (100.00)  1131 (69.90)  487 (30.10) |
| Commuting winter 2001  Passive commuters  Active commuters | 1639 (100.00)  1301 (79.40)  338 (20.60) |
| Commuting summer 2007  Passive commuters  Active commuters  Commuting winter 2007  Passive commuters  Active commuters | 1458 (100.00)  1042 (71.50)  416 (28.50)  1501 (100.00)  1233 (82.10)  268 (17.90) |
| Commuting summer 2011  Passive commuters  Active commuters  Commuting winter 2011  Passive commuters  Active commuters | 1348 (100.00)  1010 (74.90)  338 (25.10)  1352 (100.00)  1141 (84.40)  211 (15.60) |
| Commuting summer 2018  Passive commuters  Active commuters  Commuting winter 2018  Passive commuters  Active commuters | 1232 (100.00)  926 (75.20)  306 (24.80)  1232 (100.00)  1030 (83.60)  202 (16.40) |

**Supplementary Table 2**. Descriptive statistics of the continuous variables reflecting physical activity in adulthood (2001-2018/2020) (N = 961-1665)

| Variable | Min-Max | Mean (SD) | N |
| --- | --- | --- | --- |
| Self-reported LTPA  Year 2001 | 5-15 | 8.73 (1.91) | 1665 |
| Year 2007 | 5-15 | 8.76 (1.78) | 1495 |
| Year 2011 | 5-15 | 8.96 (1.86) | 1340 |
| Year 2018 | 5-15 | 8.90 (1.89) | 1335 |
| Pedometer measurements |  |  |  |
| Year 2007  Daily steps | 1501.57-22878.86 | 7801.95 (2966.49) | 1059 |
| Daily aerobic steps | 0.00-14560.33 | 2095.00 (2078.31) | 1059 |
| Daily steps (weekdays) | 1593.20-24364.40 | 7935.09 (3165.13) | 1025 |
| Daily aerobic steps (weekdays) | 0.00-14330.80 | 1892.76 (2082.04) | 1025 |
| Daily steps (weekends) | 752.00-26929.00 | 7426.68 (3559.63) | 1016 |
| Daily aerobic steps (weekends) | 0.00-18560.00 | 2613.53 (2906.48) | 1016 |
| Year 2011^1^  Daily steps | 1424.43-20798.80 | 8139.85 (3150.70) | 1088 |
| Daily aerobic steps | 0.00-14063.71 | 1958.34 (2139.78) | 1088 |
| Daily steps (weekdays) | 1314.40-22407.75 | 8281.73 (3360.16) | 1065 |
| Daily aerobic steps (weekdays) | 0.00-15377.80 | 1833.09 (2151.50) | 1065 |
| Daily steps (weekends) | 742.00-26133.00 | 7761.84 (3797.20) | 1028 |
| Daily aerobic steps (weekends) | 0.00-23937.00 | 2329.68 (3025.15) | 1028 |
| Accelerometer measurements  Year 2018/2020  Daily steps (week) | 1675.00- 22984.57 | 8584.84 (3042.17) | 961 |
| Daily aerobic steps (week)  Daily steps (weekdays) | 0.00-12943.14  1677.50-24069.40 | 1460.37 (1857.94)  8739.34 (3284.32) | 961  961 |
| Daily aerobic steps (weekdays)  Daily steps (weekends)  Daily aerobic steps (weekends) | 0.00-14305.00  1123.00-27789.50  0.00-15945.00 | 1350.68 (1835.31)  8246.14 (3710.90)  1757.08 (2712.28) | 961  961  961 |

**Supplementary Table 3**. Descriptive statistics of the variables adjusted for in the analyses (N = 1206-2436)^1^

| Variable | Min-Max | N (%) / Mean (SD) |
| --- | --- | --- |
| Gender  Women  Men | 1  2 | 2436 (100.0)  1247 (51.2)  1189 (48.8) |
| Age (y) | 9-18 | 2436 (100)/ 13.3 (3.31) |
| Education of participant's mother (1983)^1^  Primary school or below  Secondary school  Tertiary education | 1-3  1  2  3 | 1851 (100)  1499 (81.00)  237 (12.80)  115 (6.20) |
| Education of participant’s father (1983)  Primary school or below  Secondary school  Tertiary education | 1-3  1  2  3 | 1609 (100)  1277 (79.40)  213 (13.20)  119 (7.40) |
| Income of participant’s childhood family (1980)^2^ | 1-8 | 2330 (100)/ 4.80 (1.98) |
| Participant’s childhood living area (1980) | 1-4 | 2425 (100) |
| City centre | 1 | 259 (10.70) |
| Suburb | 2 | 906 (37.40) |
| Rural community | 3 | 637 (26.30) |
| Dispersed settlement area | 4 | 623 (25.70) |
| Participant’s educational status (2001) | 1-3 | 1521 (100) |
| Secondary education institution | 1 | 1106 (72.70) |
| Lower academic studies/ degree | 2 | 161 (10.60) |
| Upper academic degree | 3 | 254 (16.70) |
| Participant’s living area (2001) | 1-4 | 1742 (100) |
| City centre | 1 | 290 (16.60) |
| Suburb | 2 | 778 (44.70) |
| Rural community | 3 | 325 (18.70) |
| Dispersed settlement area | 4 | 349 (20.00) |
| Participant’s educational status (2007) | 1-3 | 1414 (100.00) |
| Secondary education institution | 1 | 939 (66.40) |
| Lower academic studies/ degree | 2 | 232 (16.40) |
| Upper academic degree | 3 | 243 (17.20) |
| Participant’s income (2007) | 1-8 | 1480 (100)/ 3.59(1.60) |
| Participant’s living area (2007) | 1-4 | 1527 (100) |
| City centre | 1 | 196 (12.80) |
| Suburb | 2 | 731 (47.90) |
| Rural community | 3 | 304 (19.90) |
| Dispersed settlement area | 4 | 296 (19.40) |
| Participant’s educational status (2011) | 1-3 | 1206 (100.00) |
| Secondary education institution | 1 | 741 (61.40) |
| Lower academic studies/ degree | 2 | 239 (19.80) |
| Upper academic degree | 3 | 226 (18.70) |
| Participant’s income (2011) | 1-13 | 1358 (100)/ 7.39 (3.05) |
| Participants’ living area (2011) | 1-4 | 1395 (100.00) |
| City centre | 1 | 176 (12.60) |
| Suburb | 2 | 637 (45.70) |
| Rural community | 3 | 270 (19.40) |
| Dispersed settlement area | 4 | 312 (22.40) |
| Participant’s educational status (2018) | 1-3 | 1308 (100.00) |
| Secondary education institution | 1 | 761 (58.20) |
| Lower academic studies/ degree | 2 | 288 (22.00) |
| Upper academic degree | 3 | 259 (19.80) |
| Participant’s income (2018) | 1-21 | 1377 (100) /9.14 (4.58) |
| Participant’s distance (km) from home to workplace (2018) | 0-417 | 1240 (100.0) / 19.22 (30.89) |

^1^Higher scores reflect higher educational level

^2^Higher scores reflect higher income level

**Supplementary Table 4.** Availability and consistency of covariates and outcome variables in the four follow-up studies

|  | Follow-up year | |  |  |  |
| --- | --- | --- | --- | --- | --- |
| Variable | 2001 | 2007 | | 2011 | 2018 |
|  |  |  | |  |  |
| Education level | CNCR | CNCR | | CNCR | CNCR |
| Income level | Suppl. 2007 | CNCR^1^ | | CNCR^1^ | CNCR^1^ |
| Living area | CNCR | CNCR | | CNCR | Suppl. 2011 |
| Distance (km) from home to work | NA | NA | | NA | CNCR |
|  |  |  | |  |  |
| Commuting summer | CNCR | CNCR | | CNCR | CNCR |
| Commuting winter | CNCR | CNCR | | CNCR | CNCR |
| LTPA | CNCR | CNCR | | CNCR | CNCR |
|  |  |  | |  |  |
| Daily steps (week) | NA | CNCR PM | | CNCR^2^ PM | CNCR^2^ AG |
| Daily aerobic steps (week) | NA | CNCR PM | | CNCR^2^ PM | CNCR^2^ AG |
| Daily steps (weekends) | NA | CNCR PM | | CNCR^2^ PM | CNCR^2^ AG |
| Daily aerobic steps (weekends) | NA | CNCR PM | | CNCR^2^ PM | CNCR^2^ AG |
| Daily steps (weekdays) | NA | CNCR PM | | CNCR^2^ PM | CNCR^2^ AG |
| Daily aerobic steps (weekdays) | NA | CNCR PM | | CNCR^2^ PM | CNCR^2^ AG |
|  |  |  | |  |  |

CNCR = concurrent and consistent data available, Suppl. = supplemented by data from given follow-up, NA = not available, PM = measured by the Omron pedometer, AG = measured by the ActiGraph accelerometer

^1^Income variable is classified as 1: €0-10000, 2: €10001-20000, …, 6: €50001-60000, 7: > €60000

^2^Notice the change of device between follow-ups

^3^A few deficiencies occured: income level in 2001 needs to be supplemented by the data of next the follow-up (2007); income data for all years is re-classified to obtain an identical covariate for each follow-up; adulthood living area in 2018 is supplemented from the previous follow-up (2011)

**Supplementary Table 5**. Childhood commuting to school in 1980 predicting adulthood responses in 2001-2018 in a multilevel modelling framework

| Variable | B | | SE | | OR | | *p* | | | 95%CI | | N_obs_ | N_ind_ |
| --- | --- | --- | --- | --- | --- | --- | --- | --- | --- | --- | --- | --- | --- |
| Active commuting, summertime | .21 | .17 | | 1.20 | | .22 | | .88–1.77 | | | 5499 | | 1917 |
| Active commuting, wintertime | -.02 | .25 | | .97 | | .93 | | .53–1.81 | | | 5563 | | 1926 |
|  |  | |  | |  |  | | | |  | |  |  |
|  | B | | SE | | β | | *p* | | | 95%CI | | N_obs_ | N_ind_ |
| LTPA | .28 | .07 | | .07 | | | <.001 | | .03–.11 | | 5663 | | 1933 |
| Daily steps (week) | 195.5 | 149.8 | | .03 | | | .19 | | -.02–.08 | | 3025 | | 1472 |
| Daily aerobic steps (week) | 225.9 | 95.8 | | .05 | | | .02 | | .01–.09 | | 3025 | | 1472 |
| Daily steps (weekends) | 317.0 | 172.4 | | .04 | | | .07 | | -.00–.08 | | 2922 | | 1452 |
| Daily aerobic steps (weekends) | 300.5 | 132.4 | | .05 | | | .02 | | .01–.09 | | 2922 | | 1452 |
| Daily steps (weekdays) | 161.4 | 160.1 | | .02 | | | .31 | | -.02–.07 | | 2973 | | 1464 |
| Daily aerobic steps (weekdays) | 216.5 | 94.4 | | .05 | | | .03 | | .01–.09 | | 2973 | | 1464 |

B = unstandardized regression coefficients, SE = standard error, OR = odds ratio, β= standardized regression coefficient, 95%CI = confidence intervals of standardized coefficient or odds ratio, N_obs_ = number of observations, N_ind_= number of participants

**Supplementary Table 6**. Childhood commuting to school in 1980 predicting adulthood responses in 2001-2018 in a multilevel modelling framework adjusting for covariates

| Variable | B | SE | OR | *p* | 95%CI | N_obs_ | N_ind_ |
| --- | --- | --- | --- | --- | --- | --- | --- |
| Active commuting, summer | -.16 | .25 | .84 | .51 | .50–1.41 | 3026 | 1038 |
| Active commuting, winter | -.21 | .33 | .77 | .52 | .35–1.70 | 3050 | 1041 |
|  |  |  |  |  |  |  |  |
|  | B | SE | β | *p* | 95%CI | N_obs_ | N_ind_ |
| LTPA | .11 | .11 | .03 | .32 | -.03–.08 | 3090 | 1038 |
| Daily steps (week) | -15.9 | 218.6 | -.00 | .94 | -.07–.06 | 1825 | 905 |
| Daily aerobic steps (week) | -60.4 | 135.1 | -.01 | .65 | -.07–.05 | 1825 | 905 |
| Daily steps (weekends) | -57.1 | 242.8 | -.01 | .81 | -.07–.05 | 1773 | 893 |
| Daily aerobic steps (weekends) | -73.5 | 185.0 | -.01 | .69 | -.07–.05 | 1773 | 893 |
| Daily steps (weekdays) | 28.1 | 236.3 | .00 | .91 | -.06–.07 | 1798 | 899 |
| Daily aerobic steps (weekdays) | -30.7 | 134.9 | -.01 | .82 | -.07–.05 | 1798 | 899 |

B = unstandardized regression coefficients, SE = standard error, OR = odds ratio, β= standardized regression coefficient, 95%CI = confidence intervals of standardized coefficient or odds ratio, N_obs_ = number of observations, N_ind_= number of participants

**Supplementary Table 7.** Attrition examinations in the questionnaire data 2001-2018^1^

| Factor (level) | OR | *p*^2^ | 95%CI (lower) | 95%CI (upper) |
| --- | --- | --- | --- | --- |
| Age (year) | 1.19 | <.001 | 1.13 | 1.26 |
| Sex (ref: woman) |  |  |  |  |
| Man | .48 | <.001 | .35 | .68 |
| Commuting to school in 1980 (ref: passive) |  |  |  |  |
| Active | 2.05 | <.001 | 1.39 | 3.04 |
| Education of participant’s mother (ref: primary school or below) |  |  |  |  |
| Secondary education | .79 | .40 | .46 | 1.37 |
| Tertiary education | .36 | .02 | .16 | .84 |
| Education of participant’s father (ref: primary school or below) |  |  |  |  |
| Secondary education | .38 | <.001 | .22 | .65 |
| Tertiary education | 1.22 | .62 | .55 | 2.75 |
| Family income (ref: lowest class < 15,000 mk) |  |  |  |  |
| Level 2 | 2.11 | .17 | .73 | 6.09 |
| Level 3 | 2.06 | .15 | .76 | 5.56 |
| Level 4 | 2.76 | .04 | 1.03 | 7.37 |
| Level 5 | 2.86 | .04 | 1.07 | 7.65 |
| Level 6 | 3.25 | .02 | 1.25 | 8.44 |
| Level 7 | 3.96 | .01 | 1.46 | 10.74 |
| Level 8 | 6.15 | .001 | 2.00 | 18.92 |
| Living area (ref: city centre) |  |  |  |  |
| Suburb | .86 | .63 | .48 | 1.56 |
| Rural community | .84 | .58 | .46 | 1.55 |
| Dispersed settlement area | .86 | .66 | .44 | 1.68 |
| Follow-up year (ref: 2001) |  |  |  |  |
| 2007 | .39 | <.001 | .31 | .50 |
| 2011 | .23 | <.001 | .18 | .29 |
| 2018 | .25 | <.001 | .20 | .32 |

Ref. = reference level, OR = Odds Ratio

^1^The sample size of the baseline population from 1980 is 2,436, of which we have full childhood covariate pattern of

1,477 participants

^2^A significant *p* value (*p* < .05) suggests that the given factor is associated with dropping out / staying in the study

across the follow-ups

**Supplementary Table 8.** Attrition examinations in the device-based data 2007-2018^1^

| Factor (level) | OR | *p*^2^ | 95%CI (lower) | 95%CI (upper) |
| --- | --- | --- | --- | --- |
| Age (year) | 1.13 | <.001 | 1.09 | 1.18 |
| Sex (reference level woman) |  |  |  |  |
| Man | .46 | <.001 | .36 | .59 |
| Commuting to school in 1980 (ref: passive) |  |  |  |  |
| Active | 1.47 | .01 | 1.09 | 1.98 |
| Education of participant’s mother (ref: primary school or below) |  |  |  |  |
| Secondary education | 1.08 | .71 | .71 | 1.65 |
| Tertiary education | .51 | .04 | .26 | .98 |
| Education of participant’s father (ref: primary school or below) |  |  |  |  |
| Secondary education | .52 | .002 | .34 | .79 |
| Tertiary education | .93 | .82 | .50 | 1.73 |
| Family income (ref: lowest class < 15 000 mk) |  |  |  |  |
| Level 2 | 1.45 | .38 | .63 | 3.33 |
| Level 3 | 1.31 | .50 | .60 | 2.88 |
| Level 4 | 1.85 | .12 | .85 | 4.02 |
| Level 5 | 1.73 | .17 | .80 | 3.74 |
| Level 6 | 2.21 | .05 | 1.00 | 4.51 |
| Level 7 | 1.86 | .12 | .85 | 4.07 |
| Level 8 | 3.46 | .01 | 1.44 | 8.33 |
| Living area (ref: city centre) |  |  |  |  |
| Suburb | .62 | .03 | .40 | .96 |
| Rural community | .70 | .09 | .46 | 1.06 |
| Dispersed settlement area | .80 | .32 | .51 | 1.25 |
| Follow-up year (ref: 2007) |  |  |  |  |
| 2011 | 1.09 | .35 | .91 | 1.32 |
| 2018 | .76 | .01 | .63 | .92 |

Ref. = reference level, OR = Odds Ratio

^1^The sample size of the baseline population from 1980 is 2,436, of which we have full childhood covariate pattern of

1,477 participants

^2^A significant *p* value (*p* < .05) suggests that the given factor is associated with dropping out / staying in the study

across the follow-ups
